# Perceptions of quality and the integrated delivery of family planning with childhood immunisation services in Kenya and Uganda

**DOI:** 10.1371/journal.pone.0269690

**Published:** 2022-06-06

**Authors:** Jessie K. Hamon, Jenna Hoyt, Shari Krishnaratne, Ariko Angela Barbra, Job Morukileng, Nathaly Spilotros, Miriam Mbembe, Seth Marcus, Jayne Webster

**Affiliations:** 1 Department of Disease Control, London School of Hygiene &Tropical Medicine, London, United Kingdom; 2 International Rescue Committee Uganda, Kampala, Uganda; 3 Uganda Public Health Fellowship Program, Ministry of Health, Kampala, Uganda; 4 International Rescue Committee US, New York, NY, United States of America; 5 World Vision International, Nairobi, Kenya; 6 World Vision US, Monrovia, California, United States of America; University of Leeds, UNITED KINGDOM

## Abstract

The integration of family planning (FP) with childhood immunisations is considered a promising approach to addressing postpartum women’s unmet need for FP in resource limited settings. This study set out to examine client and health provider perceptions of the quality of FP services that were integrated with childhood immunisations in Kenya and Uganda. Semi-structured interviews with clients (n = 30) and health providers (n = 27) were conducted in 16 rural health facilities. Interviews centred on the respondents’ experiences receiving/delivering FP services, their interactions with providers/clients, and their views on the quality of FP services. Client and provider perceptions of quality were compared through a thematic analysis of interview transcripts, and findings were synthesised using Jain and Hardee’s revised FP Quality of Care Framework. Using audit data, health facility characteristics and resources were also summarised through descriptive statistics to contextualise the qualitative findings. The dignity and respect experienced by clients was central to the respondents’ perceptions of quality. These two dimensions were not conceptualised as distinct facets of quality, but were instead perceived to be a product of the 1) access to needed services, 2) choice of contraceptives, 3) interpersonal communication, 4) information, and 5) confidentiality afforded to clients. Additionally, clients and providers alike believed that the integration of FP services with childhood immunisations had a positive effect on clients’ access to needed services and on the confidentiality they experienced in a context where modern contraceptive use was stigmatised and where a lack of support from some husbands impeded access to FP services. Understanding clients’ and providers’ conceptualisation of quality is critical to the design of high quality and client-centred integrated FP services.

## Introduction

The demand for family planning (FP) and childhood immunisations is to a large extent a function of clients’ willingness to return to a health facility or provider multiple times over several months or even years. Empirical research points to this willingness being heavily influenced by clients’ perception of the quality of services [1–5], which can differ substantially from technical or clinical indicators of quality [3, 6]. Despite a growing body of evidence that suggests that combining the delivery of FP services with childhood immunisations could help reduce the unmet need for FP among postpartum women in resource limited settings [7], little is known about the quality of integrated FP services.

Evidence from studies that have examined the integration of FP with other health services suggests that integration can have a positive impact on the quality of FP services in certain settings [8–13]. Additionally, a recent study of FP services that were integrated into primary health care delivery in South Africa revealed that communities and providers broadly defined the quality of FP services in terms of the provision of contraceptives, providers’ attitudes and contraceptive use outcomes, and that providers believed integration negatively impacted FP clients’ experiences [14]. In this context, the factors perceived to influence the quality of FP services included the providers’ technical capacity and time constraints (in part due to staff shortages), the facility’s infrastructure, as well as the interpersonal relations and information exchange between clients and providers.

However, what is not yet clear is how clients’ and providers’ perceptions of the quality of FP services that are integrated with childhood immunisations vary across different communities and health system contexts. To date, only one study has directly investigated clients’ and providers’ perspectives of the quality of FP services that are integrated with childhood immunisations [15]. That is, in Liberia, clients’ perceptions of quality were found to be influenced by: 1) the explanations they received from providers, 2) the providers’ interpersonal skills, 3) the privacy they experienced, 4) the availability of contraceptives, and 5) the providers’ respect of their choices. In this same study, providers reportedly felt that service integration had improved their interactions with clients, their record keeping systems, and the teamwork among FP and immunisation providers. They also emphasised that it had increased their workload and was at times challenging due to clients’ resistance to FP use and commodity shortages in health facilities. Additionally, very little attention has been paid to the differences between clients’ and providers’ perceptions of quality, despite evidence that suggests important discrepancies in how they view and experience integrated FP services and conceptualise the idea of reproductive quality of care [16, 17]. For example, a case study of integrated FP services in India revealed that whilst almost all providers interviewed reported offering contraceptives to their clients, fewer than 5% of clients said they received FP information or services during their latest visit [18].

To address these gaps, a qualitative study was carried out between August and November 2019 to compare client and provider perceptions of the quality of FP services that were integrated with childhood immunisations in Kenya and Uganda. This study was conducted as part of a multi-faceted process evaluation conducted in Malawi, Benin, Kenya and Uganda, which interrogated the pathways to outcomes of an NGO-supported intervention that integrated FP services with childhood immunisations in routine outreach clinics and fixed health facilities.

## Methods

A constructivist approach to qualitative research was used to examine and compare client and provider perceptions of the quality of FP services and to ascertain the dimensions of service delivery influencing their perceptions. Throughout the study, the researchers engaged in a reflexive process, systematically examining the context in which knowledge was constructed, to ensure that the findings were a reflection of the respondents’ perceptions rather than their own. The study’s methods and key findings are conveyed here according to the Standards for Reporting Qualitative Research (SRQR) [19]. Additionally, information regarding the ethical, cultural, and scientific considerations specific to inclusivity in global research is included in the S1 File.

### Study sites

The study was carried out in rural health facilities across two sites: Kenya’s West Pokot county and Uganda’s Karamoja sub-region. Although a study comparing perspectives of quality across all four countries where the process evaluation was conducted would have been ideal, inconsistencies and differences in the intervention’s implementation rendered it unfeasible.

According to the latest demographic and health surveys, the unmet need for FP was 20.8% in the Rift Valley region of Kenya (which includes West Pokot) and 19.7% in Karamoja, whilst the use of modern contraceptives among women age 15–49 in these same areas was 13.3% (married) and 6.5% (married and unmarried) respectively [20, 21]. Additionally, the proportion of children age 12–23 months receiving all basic vaccinations was 74.0% in the Rift Valley region and 73.0% in Karamoja [20, 21].

In both sites, the integrated delivery of FP services was part of a wider intervention aimed at improving access to, and use of, modern contraceptive methods among postpartum women. This intervention was supported by non-governmental organisations working in partnership with the ministries of health, and broadly involved two core components. First, community-based FP sensitisation activities were carried out via religious leaders, community leaders, and community actors. And second, FP services were provided to women seeking childhood immunisations across 25 health facilities in West Pokot and 17 health facilities in Karamoja (of which three were faith-based facilities). Depending on the health facility’s organisation and staffing level, FP services and childhood immunisations were either delivered by a single provider or by two separate providers on the same day. In most of these health facilities, FP services consisted of focused messaging, individual counselling on the use of modern contraceptive methods, and the provision of contraceptives upon request (e.g., injections, implants, condoms, pills, and intra-uterine devices). However, in health facilities characterised as faith-based, FP counselling focused exclusively on the use of natural contraceptive methods (e.g., fertility awareness) and modern contraceptives were not provided.

### Data collection

Empirical data were collected in nine health facilities in West Pokot and in seven health facilities in Karamoja, of which three were characterised as faith-based. Maximum variation sampling was used in the selection of these health facilities based on the intervention’s monitoring data [22]. Indicators considered in the selection of facilities included their usual staffing levels and FP client load, and whether the intervention was perceived by implementers to be performing well (or less well). Additionally, all three faith-based facilities in Karamoja were included in the study to ensure that differences between faith-based and non-faith-based facilities were captured.

Semi-structured interviews (SSIs) were conducted with clients and their FP providers in each of the selected health facilities by trained local interviewers. Both male and female interviewers were involved in the data collection. However, the interviewers’ gender did not have an observable influence on the interviews. Interviews were carried out in Pokot, Karamojong or English depending on the respondents’ preferred language in a space away from the health facility (at times under a nearby tree) where the confidentiality of respondents could be preserved. The SSI guide used to interview clients and providers centred on 1) their experiences receiving/delivering FP services that were integrated with childhood immunisations, 2) their interactions with providers/clients, and 3) their views on the quality of the FP services they received/delivered. Providers were also asked to explain what aspects of FP services they believed were the most and least important to clients.

Respondents were recruited using convenience sampling. Clients were approached by the interviewer upon exit from the health facility and those found eligible were invited to participate in the study. Eligible clients were 18 years or older, had attended the childhood immunisation services on the day of the interview, and had either received the integrated FP services on the same day or on a previous day since the birth of their youngest child. The inclusion of clients under 18 years of age was considered prior to the start of the study. However, almost all clients seeking FP services in the selected facilities were known to be 18 years or older. Thus, interviewing a small number of this more vulnerable group of clients was not deemed warranted for the purposes of this study. Also, eligible clients who self-reported as having an ill child on the day of the interview were excluded from participating in the study. All providers who delivered FP services on the day of the interview or who were present and usually provided FP services in the selected facilities were recruited.

Additionally, an audit was conducted with support from facility staff to supplement the qualitative data from SSIs. Using a structured questionnaire, data on the health facilities’ characteristics and the resources available on the day of the interviews were captured. Specifically, the audit focused on the facilities’ staff levels and training, inventories of FP and immunisation commodities, and their overall infrastructure.

### Data management and analysis

SSIs were audio recorded and transcribed *verbatim*. Where needed, transcripts were translated from Pokot and Karamojong into English. Anonymised transcripts were then imported into Nvivo 12 and coded around a framework, which consisted of 1) beliefs and knowledge of FP, 2) cultural, economic and religious contexts of the communities, 3) structural and process elements of FP quality of care, 4) and the effect of service integration on the quality of FP services. Coding was completed inductively with additional themes and sub-themes added when relevant. Thematic analysis was subsequently carried out, through which major and minor themes were scrutinised across study sites, and client and provider perceptions were compared to determine how they aligned and/or differed. This process was performed iteratively by two researchers (JKH and JH) to enhance the rigour of the analysis. Additionally, facility audit data were double entered from paper forms into EpiData, imported into STATA 16 for analysis, and facility characteristics were summarised using descriptive statistics.

Ultimately, results were synthesised using Jain and Hardee’s revised FP Quality of Care Framework to enable a more rigorous examination and summary of the study’s findings [23]. This framework was selected because it builds on widely recognised quality of care frameworks [24, 25] and frames the quality of FP services using a rights-based approach. It conceptualises quality in terms of four structural elements (the choice of contraceptive methods, the availability of competent providers, the availability of space to ensure audio and visual privacy, and the availability of appropriate constellation of reproductive health services) and two process elements (the appropriate information exchange with clients and the interpersonal relations between clients and providers).

### Ethics

Ethical approval for this study was obtained from the Amref Ethics and Scientific Review Committee in Kenya, the Mildmay Uganda Research Ethics Committee in Uganda, and the London School of Hygiene & Tropical Medicine ethics committee in the United Kingdom. Written informed consent was also obtained from all respondents prior to their involvement in the study.

## Results

### Respondent characteristics

A total of 57 SSIs were conducted across 16 health facilities. In all, 30 clients and 27 providers were interviewed. A summary of the respondents’ characteristics is provided in Table 1.

**Table 1:** Summary of the respondents’ characteristics.

Characteristics	West Pokot, Kenya	Karamoja, Uganda
***Clients***		
Average age of clients in years	24.5 (range 18–37)	28.7 (range 20–39)
Proportion of clients who received both services on the interview day	13/18 (72%)	12/12 (100%)
Proportion of clients who attended a faith-based facility	0/18 (0%)	6/12 (50%)
***Providers***		
Average age of providers in years	35.8 (range 25–46)	23.7 (range 22–54)
Proportion of male providers	7/15 (47%)	1/12 (8%)
Proportion of providers working in faith-based facilities	0/15 (0%)	5/12 (42%)

### Health facility characteristics

As detailed in Table 2, all health facilities included in the study had electricity and were considered to have adequate space for the integrated delivery of FP services. In West Pokot, most facilities were staffed by a single provider on the day of the interview who provided both FP and immunisation services to clients. In contrast, FP and immunisation services were generally delivered by seperate providers in Karamoja, where almost all facilities had multiple providers. Additionally, in West Pokot, all facilities offered modern contraceptives; however, commodity shortages were widespread. Conversely, in Karamoja, most contraceptives were readily available in all non-faith-based facilities where modern contraceptives were offered.

**Table 2:** Summary of health facility characteristics.

Characteristics	West Pokot, Kenya	Karamoja, Uganda
Proportion of facilities with electricity	9/9 (100%)	7/7 (100%)
Average number of rooms per facility	8 (range 4–19)	10 (range 4–16)
Average number of providers working in the facility*	6.8 (range 1–44)	4.1 (range 1–7)
Proportion of facilities staffed with only one provider on the interview day	6/9 (67%)	1/7 (14%)
Average number of providers per facility trained to deliver FP services	1.8 (range 1–4)	2.9 (range 2–5)
Proportion of facilities where both services were provided by a single provider	9/9 (100%)	1/7 (14%)
Proportion of facilities that offered modern contraceptives to clients**	9/9 (100%)	4/7 (57%)
• Proportion with contraceptive pills in stock	6/9 (67%)	4/4 (100%)
• Proportion with implants in stock	5/9 (56%)	4/4 (100%)
• Proportion with contraceptive injections in stock	7/9 (78%)	4/4 (100%)
• Proportion with intra-uterine devices in stock	2/9 (22%)	3/4 (75%)

* One facility in West Pokot was a large sub-district facility with 44 providers.

**Modern contraceptives were only offered in non-faith-based facilities.

### Community contexts

In both sites, the community contexts in which clients sought services were characterised by a lack of support for modern contraceptive use. This was partly fuelled by concerns about physical side effects, but also by myths and misinformation linking contraceptive use to infertility and illness. For some clients, this translated into a fear of being stigmatised for using modern contraceptives and a need for confidential or covert family planning services. In Karamoja, the lack of support for modern contraceptives was also driven by a strong preference for natural methods and providers reported that explaining the benefits of modern contraceptive use required repeat counselling sessions. Despite this lack of community support for contraceptive use, in both sites, clients and providers said that women recognised the health and economic benefits of using contraceptive methods to assist with healthy spacing and limiting family sizes.

*“I fear the existing stigma where our community is negative about family planning*. *Attempts have been made to advocate for these modern methods but end up in quarrels within families*. *Nonetheless*, *there is a gradual shift to embrace the methods by some women who use their rights to stealthily join family planning methods*. *Such women see the burden they face and decide to forcefully join family planning to get out from the bondage of rapid-child-bearing*.*”* Client 01, Karamoja, Uganda

*“You find that in the community there are so many challenges*, *like you find that a mother has come for the family planning service but when she goes back [to her community]*, *she is intimidated by others… Other mothers talk like*, *‘why have you gone for family planning*, *you see that thing is going to kill you*, *you will not produce again’*.*”* Provider 07, Karamoja, Uganda

Additionally, a lack of support for modern contraceptives among husbands was reported in both sites and was viewed as a further impediment to contraceptive use. In Karamoja, clients and providers reported fearing husbands’ disapproval of contraceptive use. For example, clients were afraid to speak out in support of contraceptive use in the community due to fears of retaliation from unsupportive husbands. Likewise, in West Pokot, husbands were perceived to distrust women who use contraceptives. Providers in both sites explained that some clients pursue FP services covertly as a result of unsupportive husbands, such as seeking services at the end of the day when the facility is empty to avoid being seen. However, a few clients and providers in West Pokot said that some husbands supported contraceptive use and were involved in the decision to use contraceptives.

*“I fear husbands that do not want their wives to join family planning*. *If any of these negative men learns that I am the one influencing his wife for family planning*, *it will be war and hatred*.*”* Client 12, Karamoja, Uganda

*“Mostly when you ask* [the women], *they tell you that the husband believes that when a woman uses family planning it’s related to prostitution*, *so* [a woman] *fears when the husband finds out that she is using family planning things will not go well”* Provider 07, West Pokot, Kenya

### Perceptions of the quality of integrated FP services

When clients were asked about the quality of the FP services they received, they generally likened a positive experience to feeling respected. Similarly, providers commonly equated the quality of the FP services that they delivered alongside childhood immunisations with the dignity and respect afforded to clients. In the studied context, respect was described by respondents in terms of the 1) access to needed services, 2) choice of contraceptives, 3) interpersonal communication, 4) information, and the 5) confidentiality afforded to clients. This represents a departure from Jain and Hardee’s revised FP Quality of Care Framework, in which ‘treating clients with dignity and respect’ is conceptualised separately from other elements of quality such as the choice of contraceptives, and relates primarily to the availability of skilled personnel and interpersonal relations.

#### Access to needed services

In both sites, clients equated being respected to receiving the services they needed and accessing them in a timely manner. On the day of their interview, most clients stated that the wait time in the facility was short or of an acceptable length. In general, they reported feeling respected when wait times in the facility were short and when contraceptives and FP providers were available. Additionally, understaffing and contraceptive shortages were seen as disrupting clients’ access to FP services in both sites, which was interpreted by clients as a lack of respect. This was echoed by providers in both sites who demonstrated a clear understanding of clients’ views around this subject.

“*They gave high respect when I arrived*. *They humbly greeted and showed me where to sit*. *Then they asked what service I needed in the unit*. *They also attend to patients faster*. *All these are signs of respect and esteem to patients”*. Client 06, Karamoja, Uganda

*“First when* [the clients] *arrive*, *we treat them with respect and after counselling if there is* [a client] *who accepts* [a contraceptive], *we will help her with urgency* [and] *by so doing*, *she will see that she has been respected because she has not waited until she became tired*. *She was assisted in time*.*”* Provider 12, West Pokot, Kenya

Although respondents generally perceived the integration of FP services with childhood immunisations as improving women’s access to needed services by eliminating the need to attend the health facility on separate days, it was perceived to undermine clients’ access in other ways. For instance, providers in West Pokot pointed out that wait times in facilities were prolonged by service integration when a single provider was tasked with delivering both services to all clients as integration increased providers’ workload. Other providers also highlighted that whilst service integration may have improved some women’s access to services, it failed to have a similar effect on same day uptake of contraceptives as many clients wished to consult their husband prior to adopting a method.

*“It is advisable to integrate both services and deliver on the same day because both the mother and her child receive services the same day instead of repeated visits to health units*.*”* Client 04, Karamoja, Uganda

“[Service integration] *is a lot of work*. *Sometimes we get tired immunizing a lot of mothers*. *Then women are here waiting to be given family planning*… *Many times* [integration] *helps the client more but for the health worker it’s a lot of work…especially when you are one*, *but when you are two or three health workers it’s not an issue*.” Provider 09, West Pokot, Kenya

#### Choice of contraceptives

In Karamoja and West Pokot, providers spoke of the clients’ freedom to choose a contraceptive as central to the respect afforded to clients and in turn to the quality of services. They emphasised that FP providers should respect the clients’ choices in general, but most importantly the contraceptive they decide to use. In West Pokot, clients similarly linked the freedom to choose a contraceptive to being respected by providers. Most of these clients reported feeling respected on the day of the interview because they were given the opportunity to choose a contraceptive after having received information about different methods, and because their choices were respected by the FP provider. Conversely, in Karamoja, some clients said the FP provider chose a suitable contraceptive for them, and others who sought services in faith-based facilities reported only being counselled on natural methods. Despite having relatively less freedom of choice then clients who sought services in other health facilities, clients in faith-based facilities reported being content with the quality of services they received because their provider ultimately respected their choices.

*“Interviewer (I)*: *How did the health worker respect you*? *Respondent (R)*: *The health worker respected my decisions according to my choice and what I said and helped me*.*”* Client 09, West Pokot, Kenya

*“R*: *I was confident and happy to receive a family planning method and the nurse instructed me to strictly follow it for the benefit of child spacing*. *I*: *Who chose for you the method*? *R*: *She chose for me the method of using pills*.*”* Client 09, Karamoja, Uganda

#### Interpersonal communication

Providers perceived positive interpersonal communication as central to the dignity and respect afforded to clients, and therefore believed it to be crucial to the quality of integrated FP services that were integrated with childhood immunisations. In both sites, providers mentioned that welcoming clients and adopting a good attitude towards them were key to effective communication, which in turn helped them build relationships with clients and influenced clients’ willingness to return for follow-up services in the future. Clients in West Pokot and Karamoja similarly interpreted the way in which providers communicated as a manifestation of respect and thus as a sign of the quality of services. Examples stated by clients included whether providers spoke politely and professionally, and whether they listened attentively to clients and avoided shouting at them.

*“The only thing that matters is the counselling and the attitude of the health worker*, *if a mother comes now*, *I give her health talk and I don’t use a friendly tone like if I say*, *‘you women you should stop giving birth anyhow*.*’ You see no one would approach me later or even listen to what I would say*. *So*, *we need to have that positive attitude*.*”* Provider 05, West Pokot, Kenya

“*I was well received by health workers and interacted well with them about family planning*. *The nurse was also pleased when I told her that I had come to the unit to join family planning… They have respect and love for patients*. *I liked the way they talked to me with humility and concern*.” Client 02, Karamoja, Uganda

#### Information received from providers

Clients indicated that the information they received from providers on 1) the benefits of FP, 2) the different types of contraceptives, and 3) the possible side effects of contraceptives was an important part of the quality of the services they experienced. For example, in West Pokot, clients reported feeling respected when they received explanations and reassurance from providers about contraceptive side effects. Although providers did not likewise directly link the information they delivered to the respect experienced by clients, they believed that their knowledge and counselling skills were critical to the delivery of FP services.

“*The health worker respected me in advising me what to do*. *I was told to stay for two years without giving birth for your body to be strong because if we give birth fast then the body will be weak*.*”* Client 13, West Pokot, Kenya

*“It is the baseline of care*, *for effective provision of the service*. *The health worker must be knowledgeable and skilful in order to provide the service*, *counselling and manage the side effects*.*”* Provider 11, Karamoja, Uganda

#### Confidentiality afforded to clients

A common view among providers was that the level of confidentiality achieved when delivering FP services was key to the respect and dignity experienced by clients and to their perception of the quality of these services. According to providers, confidentiality could be achieved when FP counselling was carried out 1) with individual clients rather than in groups, 2) in a separate room away from clients seeking other health services, and 3) by keeping clients’ personal information private.

*“Actually*, *the most important thing that we are required [to do] is when you want to show someone respect* [you give them] *privacy*. *That is what even makes someone more open…Privacy is most important* [for] *family planning…Our health facility as you see*, *we lack space…all maternal and child health services are in one room*, *so we need space as a facility*.*”* Provider 11, Karamoja, Uganda

*“I*: *As you give the services*, *how do you make sure they feel respected*? *R*: *There is confidentiality*. *When I counsel a mother and she accepts a certain method there is no one that knows about it and it is not written anywhere so it is done in secrecy… It is done in a room that is private*, *no one sees or hears about it*.*”* Provider 06, West Pokot, Kenya

Additionally, in both sites, providers stated that facilitating covert contraceptive use was a way of respecting clients and thus of providing quality services. For example, in West Pokot, providers reported administering contraceptives after health facility hours to clients who wanted to avoid being seen receiving FP services. Of note, providers believed that service integration improved their ability to support clients requiring covert access to FP services because childhood immunisations provided a legitimate reason for women to visit the health facility.

*“We also respect the dignity of our client because we don’t know if the husband approved* [the use of contraceptives] *or not*. *Because every human being has a right to decide for herself…you might get that in a family the woman has seen the importance of having a planned family*, *but the man has not seen*, *so we assist her according to her wish*. *It is not good for us to expose her secrets and we also assure* [clients] *that it is a secret between them and the health worker*, *nobody else will know*.*”* Provider 12, West Pokot, Kenya

Despite seeking FP services in a community context where the use of modern contraceptives is not widely supported and thus confidentiality is critical for many clients, only a few clients spoke of confidentiality during the interviews. For example, one client stated that she felt good about her experience because she believed that the provider would not share with others the information she disclosed during her counselling session, and another was positive about her experience because the providers were willing to help clients access contraceptives without their husband’s knowledge.

## Discussion

This study aimed to examine client and provider perceptions of the quality of FP services that were integrated with childhood immunisations in Kenya’s West Pokot county and Uganda’s Karamoja sub-region. A comparison of respondents’ views and experiences revealed considerable alignment between providers’ and clients’ perceptions of quality and a shared belief that service integration improves clients’ access to FP services. In fact, the only noteworthy difference found between clients’ and providers’ perceptions was that, unlike clients, providers did not directly associate the information they provided to clients with the quality of FP services. Surprisingly, the views of respondents from West Pokot and Karamoja were also largely consistent. A partial explanation for this could be the similarities in community-level contexts between the two sites, which may be influenced by the geographical proximity between Karamoja and West Pokot, despite being located in different countries. However, an important difference between the two sites was that providers in West Pokot expressed concerns about disruptions to clients’ prompt access to needed services due to staff shortages in health facilities. This concern was not raised by providers in Karamoja, where, according to audit data, almost all facilities were staffed by multiple providers, more providers on average were trained to deliver FP services, and where FP and childhood immunisation services were more commonly delivered by two separate providers. This finding suggests that variation in staffing levels at health facilities can cause noticeable differences in the quality of integrated FP services, even when all other relevant factors (including community contexts) are found to be similar.

Overall, the dignity and respect experienced by clients was found to be central to both clients’ and providers’ perceptions of quality in the studied contexts. Although dignity and respect are recognised as key dimensions of quality FP services in several studies [5, 26], they were not reported in comparable research conducted in Liberia and Malawi that examined client and providers’ perceptions of integrated FP services [15, 27]. Additionally, rather than being conceptualised as a distinct element of quality as is often done [23, 25, 28–31], the dignity and respect experienced by clients was perceived as a product of the access to needed services, the choice of contraceptives, the interpersonal communication, the information and the confidentiality they were afforded. Interestingly, this perception held true among clients in Karamoja who attended faith-based health facilities where the choice of contraceptives was limited by the absence of modern contraceptive methods. Even though services delivered in these health facilities did not objectively meet key structural dimensions of quality set out in Jain and Hardee’s revised FP Quality of Care Framework (i.e. choice of method and the availability of appropriate constellation of reproductive health services), clients nonetheless reported being content with their experience of the services because they felt their choices (albeit limited) were respected by providers. In fact, contrary to expectations, this study did not find any significant differences between the perceptions of quality among clients who sought services in faith-based and non-faith-based facilities.

Furthermore, client-provider interactions and rapport were found to be a critical part of clients’ perceptions of quality. This is consistent with prior evaluations of the quality of FP services [5, 32, 33] and Nelson *et al’*s evaluation of the quality of integrated FP services in Liberia [15]. It also mirrors the conclusion reached by Stichler and Weiss on the quality of women’s healthcare that “women want and expect competence in their providers and evaluate this competence based on personal interactions” [17]. In particular, clients in this study felt the providers’ professionalism was key to the quality of services, both in terms of the information they received and the interpersonal communication they experienced when interacting with providers. Also, although not explicitly discussed, examples cited by respondents commonly conveyed an implicit trust of providers. For instance, providers spoke of facilitating clients’ covert use of contraceptives in communities where FP was stigmatised or when a client’s husband was unsupportive of modern contraceptive use, suggesting clients trusted their health providers. This is consistent with findings from a study of service quality and responsiveness in Zambian primary health centres [34], where trust of providers was central to perceptions of quality and where disrespectful care was seen as damaging clients’ trust in providers’ values and professionalism, ultimately resulting in some clients seeking services elsewhere. This finding has important implications for service delivery as it calls attention to the critical influence that ‘software elements’ of the health system [35], such as trust and provider behaviour, have on clients’ perceptions of quality, and consequently on their willingness to return to a health facility to seek FP services when these are integrated with childhood immunisations. As such, this study further supports the idea that looking beyond infrastructure or structural components of service delivery is critical to achieving measurably better quality of integrated health services [36–38].

## Limitations

Given the approach to data collection used in this study, it was not possible to fully mitigate social desirability biases. Clients’ perceptions of quality may have also been influenced by a Hawthorne effect. It is possible that providers delivered FP services differently on the day of the interview due to the interviewers’ presence in the facilities, which may have shaped more positively clients’ views of the quality of services. Additionally, clients’ perceptions of quality and their conceptualisation are likely to be refined through repeat exposure to services, a longitudinal approach to data collection may have revealed further insights not captured by the cross-sectional design that was adopted in this study. Finally, as this study was conducted with clients and providers in health facilities, the perceptions of non-FP users or clients who have opted to seek services elsewhere were not captured, which represents an important limitation.

## Conclusion

This study set out to examine and compare client and provider perceptions of the quality of FP services that were integrated with childhood immunisations in health facilities. Overall, the respect and dignity experienced by clients were found to be central to perceptions of quality in the studied context. Crucially, respect and dignity were not conceptualised as distinct facets of quality by respondents but were instead perceived to result from other dimensions of quality such as the confidentiality that clients experienced while seeking services. These findings demonstrate that understanding clients’ and providers’ conceptualisation of quality is critical to the design of high quality and client-centred integrated FP services.

## Supporting information

S1 File Inclusivity in global research questionnaire. (PDF)
